# Brain Imaging and Whole Blood Targeted Transcriptomic Analyses to Characterize Cerebral Infarctions in Children With Tuberculous Meningitis

**DOI:** 10.1093/infdis/jiaf399

**Published:** 2025-08-02

**Authors:** Julie Huynh, Pieter M Pretorius, Wajanat Jan, Carolina Kachramanoglou, Nhat Hoang Thanh Le, Van La Ngoc, Hai Thanh Hoang, Ny Thi Hong Tran, Tram Ngoc Pham, Thu Anh Dang Do, Dung Thi Mong Vu, Trinh Thi Bich Tram, Do Dinh Vinh, Tung Huu Trinh, Nguyen Dinh Qui, Minh Ha Thi Dang, Elena Frangou, Sierra Santana, Caitlin Muller, Suzanne T Anderson, Diana M Gibb, Nhung Thi Hong Nguyen, Nguyen Thuy Thuong Thuong, Guy Thwaites

**Affiliations:** Centre for Tropical Medicine and Global Health, Nuffield Department of Medicine, Oxford University, Oxford, United Kingdom; Oxford University Clinical Research Unit, Ho Chi Minh City, Vietnam; Oxford University Hospitals NHS Foundation Trust, Oxford, United Kingdom; Imaging Department, Imperial College Healthcare NHS Trust, London, United Kingdom; Imaging Department, Imperial College Healthcare NHS Trust, London, United Kingdom; Oxford University Clinical Research Unit, Ho Chi Minh City, Vietnam; Oxford University Clinical Research Unit, Ho Chi Minh City, Vietnam; Oxford University Clinical Research Unit, Ho Chi Minh City, Vietnam; Oxford University Clinical Research Unit, Ho Chi Minh City, Vietnam; Oxford University Clinical Research Unit, Ho Chi Minh City, Vietnam; Oxford University Clinical Research Unit, Ho Chi Minh City, Vietnam; Oxford University Clinical Research Unit, Ho Chi Minh City, Vietnam; Oxford University Clinical Research Unit, Ho Chi Minh City, Vietnam; Oxford University Clinical Research Unit, Ho Chi Minh City, Vietnam; Infectious Diseases Department, Children's Hospital 2, Ho Chi Minh City, Vietnam; Infectious Diseases Department, Children's Hospital 2, Ho Chi Minh City, Vietnam; Paediatric Department, Pham Ngoc Thach Hospital, Ho Chi Minh City, Vietnam; Medical Research Council Clinical Trials Unit at University College London, United Kingdom; Medical Research Council Clinical Trials Unit at University College London, United Kingdom; Medical Research Council Clinical Trials Unit at University College London, United Kingdom; Medical Research Council Clinical Trials Unit at University College London, United Kingdom; Medical Research Council Clinical Trials Unit at University College London, United Kingdom; Paediatric Department, Pham Ngoc Thach Hospital, Ho Chi Minh City, Vietnam; Centre for Tropical Medicine and Global Health, Nuffield Department of Medicine, Oxford University, Oxford, United Kingdom; Oxford University Clinical Research Unit, Ho Chi Minh City, Vietnam; Centre for Tropical Medicine and Global Health, Nuffield Department of Medicine, Oxford University, Oxford, United Kingdom; Oxford University Clinical Research Unit, Ho Chi Minh City, Vietnam

**Keywords:** cerebral infarctions, inflammation, matrix metalloproteinases, transcriptomic

## Abstract

We characterized cerebral infarction in children with tuberculous meningitis and explored its relationship with systemic inflammatory mediators using targeted transcriptomic analysis. Children with tuberculous meningitis had baseline magnetic resonance imaging scans and whole blood RNA sequencing for matrix metalloproteinases (MMP-8, MMP-9, TIMP-1), cytokines (IL-10, IL-1β, TNF-α, IFN-γ), and growth factors (VEGF). Overall 22 (73%) children had mild disease and 19 (63%) had cerebral infarctions, which were commonly acute (n = 9, 47%), multiple (n = 14, 74%), and bilateral (n = 12, 63%), occurring in cerebral hemispheres (n = 12, 59%), basal ganglia (n = 10, 53%), and thalamus (n = 5, 26%). Children with infarctions had significantly higher cerebrospinal fluid protein, lower cerebrospinal fluid glucose, and higher systemic MMP-8 expression.

## Teaser

Silent cerebral infarctions are common in children with tuberculous meningitis of mild severity. This study links these infarctions to elevated systemic MMP-8 expression, suggesting a role in inflammation-mediated brain injury and potential for targeted therapies.

Cerebral infarctions occur in 40% to 70% of children with tuberculous meningitis (TBM) and are strongly associated with death or poor neurodevelopmental outcomes [1, 2]. Infarcts are commonly associated with basal inflammatory exudates encasing the cerebral arteries and their perforators. The resulting vasculitis, ischemia, and infarction particularly affect the basal ganglia and internal capsules [3].

TBM is associated with the activation of macrophages and microglia and the release of proinflammatory cytokines: tumor necrosis factor α (TNF-α), interleukin 1β (IL-1β), and interferon γ (IFN-γ) [4]. The immune response is critical for pathogen containment, but excessive inflammation leads to disruption of the blood-brain barrier, increased vascular permeability, and cerebral complications that ultimately contribute to neuronal injury and long-term neurologic deficits [3]. To date, only adjunctive anti-inflammatory corticosteroids are proven to reduce mortality but with little effect on infarct incidence or neurodisability [5]. Adjunctive high-dose aspirin may reduce infarcts, although its impact on outcomes is awaiting the completion of phase 3 trials [6, 7]. A better understanding of the pathophysiology of TBM-associated infarcts is needed to develop more effective host-directed therapies. Our aim was to use brain imaging and targeted whole blood transcriptomics to characterize the inflammation associated with cerebral infarcts in children with TBM. Inflammatory mediators of interest were matrix metalloproteinases (MMPs), a family of proteolytic enzymes that degrade extracellular matrix and break down the blood-brain barrier; inflammatory cytokines, which mediate immune cell recruitment; and growth factors (eg, VEGF), which have roles in angiogenesis and vascular permeability. All have been implicated in TBM pathogenesis [4].

## METHODS

Children aged 29 days to 18 years with TBM were prospectively enrolled into the study from March 2021 to November 2022. The study was nested within an ongoing clinical trial evaluating short intensified antituberculosis therapy and adjunctive aspirin for children with TBM (SURE; ISRCTN40829906) [7]. Children were eligible if they had signs and symptoms compatible with TBM, with or without microbiological detection of *Mycobacterium tuberculosis* in the cerebrospinal fluid (CSF), and had not received >21 days of antituberculosis therapy. TBM diagnosis was retrospectively classified into definite, probable, and possible TBM according to a published uniform research case definition [8]. Demographic and clinical characteristics were recorded at enrollment, including disease severity (per a refined British Medical Research Council [MRC] grade; Supplementary Table 1). Written informed consent was obtained from all parents/carers and assent from children aged ≥12 years. The study was approved by the institutional review board at Pham Ngoc Thach Hospital and Children's Hospital 2, Ho Chi Minh City, and the Vietnam Ministry of Health.

### Brain Imaging

Brain magnetic resonance imaging (MRI) scans were obtained with standardized protocols and a 1.5-T system (Siemen and GE HealthCare Technologies). MRI sequences were as follows: axial diffusion-weighted imaging with apparent diffusion coefficient map, axial T2, axial gradient echo T2*, axial T2 fluid attenuation inversion recovery, and volumetric axial T1-weighted pre- and postgadolinium (intravenous, 0.1 mmol/kg). Volumetric sequences were 1 mm while all other sequences were 4 mm thick and evaluated by Efilm (Japan).

Baseline brain MRI was performed within ±7 days of enrollment. Additional brain MRI, performed as part of normal clinical care, were included if images were of adequate quality and followed the standardized protocol. Study imaging was performed without sedation or anesthesia and was abandoned after 2 attempts if imaging could not be tolerated.

Independent and systematic assessment of images was undertaken by 3 experienced neuroradiologists (blind to treatment and outcome) in London via a central repository. Any disagreement was discussed and final consensus reached during reporting sessions. Enlarged ventricles without cerebral volume loss was defined as hydrocephalus. Communicating or noncommunicating hydrocephalus was defined by the absence or presence of an obstructing lesion along the intraventricular CSF pathways. Meningeal enhancement was defined as confluent or nodular (meningeal tuberculoma) enhancement of meninges. Basal meningeal enhancement was defined as meningeal enhancement at 1 or more of the following locations: basal cisterns, ambient cistern, quadrigeminal cistern, prepontine cistern, cerebellopontine cistern, and suprasellar cistern. Parenchymal ring-enhancing lesions with liquified centers >3 cm were reported as tubercular abscesses. Tuberculomas were defined as nodular or ring-enhancing lesions and could be parenchymal, meningeal, or ventricular. Infarcts were classified as acute if they demonstrated restricted diffusion—specifically, hyperintense appearances on *b*1000 diffusion-weighted imaging and confirmed by decreased apparent diffusion coefficient on the apparent diffusion coefficient map. All lesions were reported in prespecified anatomic locations.

### Inflammation Assessment

Venous blood was collected in PAXgene Blood RNA tubes (PreAnalytiX) at enrollment and stored at −80 °C. RNA was manually extracted with the PAXgene Blood RNA Kit (Qiagen). DNA was digested on columns with the RNase-Free DNase Set (Qiagen), with RNA sequencing performed at the Ramaciotti Centre for Genomics and libraries generated as previously described [9].

Quality control and alignment of RNA sequencing data were performed through an in-house pipeline as used in our previous studies [9]. Gene expression counts were generated from sequencing FASTQ files. Counts were normalized for sequence depth and RNA composition with the DESeq2 package. Normalized gene expression on a predetermined 10-gene panel was extracted for the main analysis: TNF-α, IL-1β, IL-10, IFN-γ, MMP-9, MMP-10, TIMP-1, and VEGF, with neuromarkers S100B and ENO2 as noninflammatory controls.

### Data Analysis

For continuous variables, median and IQR were presented. For binary and categorical variables, number of cases and percentage were presented. All variables were predetermined before testing. Wilcoxon rank sum test and Fisher exact test were performed to compare clinical, CSF, and brain MRI features of TBM between children with and without infarcts. Comparisons of gene expression and the relationships with MRI features were descriptive. All statistical analyses were conducted in R version 4.3.0.

## RESULTS

Fifty-five children were enrolled to the study: 30 had baseline brain MRI and 25 were unable to undergo imaging (13 due to excessive patient movement; 3 because of unstable medical conditions; 3 because of COVID-19 restrictions; 2 died before imaging; 1 was withdrawn with an alternative diagnosis; and 3 had imaging performed outside the ±7-day protocol window; Supplementary Table 2). In those imaged, MRI was taken at a median 3.0 days (IQR, 1.3–4.0) after enrollment. Baseline characteristics are presented in Table 1: 22 (73%) children were male, the median age was 11.0 years (IQR, 2.5–13.1), and 7 (58%) with definite TBM were ≥11 years old (Supplementary Table 3). The majority (n = 22, 73%) had mild disease (MRC grade 1).

**Table 1.** Baseline Characteristics in Children With TBM Who Had Baseline MRI (n = 30)

	Median (IQR) or No. (%)
Patient characteristics	
Age, y	11.0 (2.5–13.1)
Female	8 (27)
BCG vaccinated	25 (83)
HIV-negative status	30 (100)
Fever	30 (100)
Fever duration, d	10 (7–18)
Glasgow coma scale	15 (15–15)
Cranial nerve palsy	4 (13)
Hemi-, para-, or tetraplegia	4 (13)
Modified BMRC grade^a^	
I	22 (73)
IIa	6 (20)
IIb	1 (3.3)
III	1 (3.3)
Extraneural TB disease	8 (27)
TBM classification	
Definite	12 (40)
Probable	7 (23)
Possible	11 (37)
CSF characteristics	
Leukocyte count, ×10^3^ cells/mL	145 (51–531)
Lymphocytes, %	82 (65–98)
Protein, g/L	1.24 (0.91–1.89)
Glucose, mmol/L	2.01 (1.59–2.65)
CSF/blood glucose ratio	0.34 (0.28–0.42)
Blood characteristics	
Leukocyte count, ×10^6^ cells/mL	10.6 (9.0–14.6)
Lymphocytes, %	25 (17–34)
Neutrophils, %	57 (44–70)
Platelets, ×10^3^/μL	402 (370–521)
Sodium, mmol/L	133.0 (127.5–135.8)
MRI findings	
Infarct	19 (63)
Laterality: bilateral	12 (63)
Chronicity	
Acute	9 (47)
Chronic	6 (32)
Both	4 (21)
No. of infarcts/patient	
1	5 (26)
2–4	8 (43)
>4	6 (32)
Location	
Cerebral hemispheres	12 (59)
Basal ganglia	10 (53)
Thalamus	5 (26)
Brainstem	4 (21)
Cerebellum	2 (12)
Other^b^	7 (59)
Hydrocephalus	18 (60)
Communicating	18/18 (100)
Tuberculoma	9 (30)
Meningeal enhancement	8 (27)
Basal enhancement	5/8 (62)
Cranial nerve enhancement	3 (10)
Tubercular abscess	0 (0)
>1 finding	22 (73)

Abbreviations: BCG, bacillus Calmette-Guerin; BMRC, British Medical Research Council; CSF, cerebrospinal fluid; MRI, magnetic resonance imaging; TB, tuberculosis; TBM, tuberculous meningitis.

^a^Modified BMRC: grade I, Glasgow coma scale of 15 and no focal neurology; grade IIa, Glasgow coma scale of 15 with focal neurology; grade IIb, Glasgow coma scale of 11–14 with or without focal neurology; grade III, Glasgow coma scale ≤10.

^b^Other: corpus callosum (n = 6) and anterior commissure (n = 1).

### MRI and Clinical Features

Of the 30 children who had baseline brain MRI, 26 (87%) had abnormal findings (Table 1). Brain infarction was present in 19 (63%), hydrocephalus in 18 (60%), tuberculoma in 9 (30%), and meningeal enhancement in 8 (27%). Cerebral infarctions were commonly acute (n = 9, 47%), multiple (n = 14, 74%), and bilateral (n = 12, 63%). The most common locations were cerebral hemispheres (n = 12, 59%), basal ganglia (n = 10, 53%), and thalamus (n = 5, 26%). Four children had infarction without other radiologic features of TBM.

As compared with children without infarcts, those with cerebral infarcts had more seizures (5 vs 0, *P* = .129), focal neurology (5 vs 1, *P* = .372), hydrocephalus (14 vs 4, *P* = .063), and extraneural disease (6 vs 2, *P* = .672; Supplementary Table 4). Infarction was not significantly associated with more severe disease, defined as MRC grades IIb and III. However, children with infarcts had significantly higher CSF protein (median, 1.7 g/L [IQR, 1.0–2.1] vs 1.1 g/L [IQR, .4–1.3]; *P* = .045) and lower CSF glucose (median, 1.8 [IQR, 1.4–2.4] vs 2.4 [IQR, 2.0–2.7]; *P* = .02).

### Inflammation and Brain Imaging

**Figure 1.**
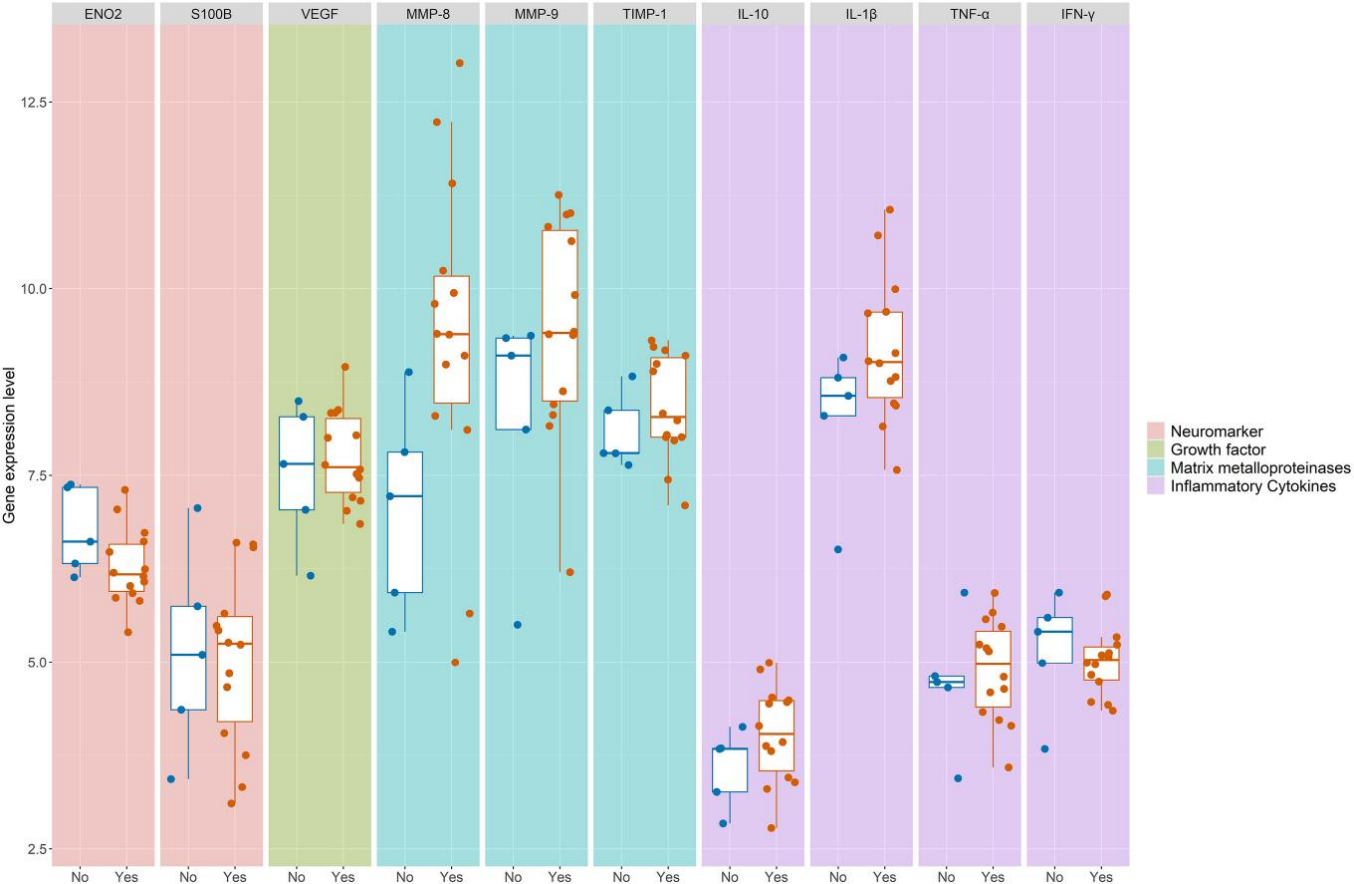
Expression of selected inflammatory mediators in the whole blood of children with infarcts and TBM infection. Nineteen children with definite, probable, or possible TBM had baseline magnetic resonance imaging and whole blood RNA sequencing for neuromarkers (ENO2, S100B), inflammatory cytokines (IL-1β, IFN-γ, TNF-α, IL-10), growth factors (VEGF), and matrix metalloproteinases (MMP-8, MMP-9, TIMP-1). Fourteen children had radiologically determined infarcts and 5 did not. Dots, individual patient data; boxes, IQR; line within box, median; whiskers, values up to 1.5 times IQR. MMP-8 expression was increased in children with cerebral infarcts. Abbreviation: TBM, tuberculous meningitis.

Whole blood RNA sequencing was performed in 19 of 30 children with brain MRI (10 definite TBM, 2 probable, and 7 possible). The median age was 7.8 years (IQR, 1.5–13.4). Selected genes were grouped into neuromarkers (ENO2, S100B) as noninflammatory controls, growth factors (VEGF), MMPs (MMP-8, MMP-9, TIMP-1), and inflammatory cytokines (IL-1β, IL-10, TNF-α, IFN-γ). MMP-8 expression was higher in the 14 children with cerebral infarcts (9.39 gene expression count; IQR, 8.47–10.16) as compared with the 5 without (7.22 gene expression count; IQR, 5.93–7.81; Figure 1). MMP-9, TIMP-1, S100B, VEGF, IL-1β, IL-10, TNF-α, and IFN-γ expression was similar in children with TBM-associated infarcts vs those without. MMP-8 expression was higher in children with hydrocephalus, but expression of other inflammatory mediators was not different (Supplementary Figure 1). There was no difference in expression of inflammatory mediator profiles in those with or without tuberculomas (Supplementary Figure 2). MMP-8 expression was higher in children with concomitant extraneural tuberculosis (Supplementary Figure 3).

## DISCUSSION

TBM remains the most serious form of tuberculosis, with associated cerebral infarcts an important cause of mortality and neurodisability. However, the pathophysiology of infarcts is poorly understood, especially in children, which limits their prevention and treatment.

We have characterized cerebral infarcts in children with TBM using MRI and explored their link with systemic inflammation. We found that cerebral infarcts were the most common CNS complication in children with TBM and that they were mostly clinically silent, occurring in children with mild disease. Additionally, the development of these cerebral infarcts may be mediated by systemic MMP-8. These findings provide important initial insights into immunopathology driving disease in TBM and for the development of patient-individualized, host-directed therapy.

Cerebral infarcts occur in 40% to 70% of children with TBM, although this figure varies by modality of neuroimaging [1, 2, 10]. In children, hydrocephalus has been reported as a more common complication (occurring in 42%–88%) [11], but this may be a result of previous studies including those with advanced disease and using computed tomography imaging, which identifies hydrocephalus accurately but may miss small infarcts [10]. It is striking that in our cohort, cerebral infarcts were more common than hydrocephalus and were not associated with disease severity, occurring mostly in children with mild disease without focal neurologic symptoms or altered sensorium. The infarcts were predominantly multiple, bilateral, and located in vascular territories of the anterior, middle, and posterior cerebral arteries, especially the basal ganglia. We do not have long-term functional and neurodevelopmental end points to evaluate the clinical consequences of these findings. Previous studies suggest that neurodevelopmental outcomes in children with radiologically confirmed infarcts were worse than those without infarcts [2, 12]. Our findings therefore have implications for prognostication and early intervention in high-risk cases of children with TBM. Long-term neurodevelopmental outcomes should be further investigated.

To better understand the immunopathology of TBM-associated cerebral infarcts, we explored the systemic expression of inflammatory mediators previously implicated in the pathogenesis of TBM. MMP expression, notably MMP-8, was higher in children with infarcts vs those without and in those with extraneural tuberculosis. Higher concentrations of MMP occur in serum than CSF [13], although its role in TBM pathogenesis is uncertain. MMP-8 (a collagenase) has the ability to break down fibronectin in the brain vascular membrane and aggrecan in brain parenchymal extracellular matrix, sharing similar substrates to the gelatinases (MMP-2 and MMP-9) [14]. High CSF MMP concentrations in children with TBM have previously correlated strongly with leptomeningeal enhancement, hydrocephalus, and poor clinical outcomes [15].

The small sample size and the inclusion of children with predominantly mild disease limit our ability to draw conclusions on the relationship between disease severity and infarcts and between systemic inflammatory mediators and neuroradiologic abnormalities. There may have been selection bias by inadvertent exclusion of children with severe disease due to clinical instability or early death; however, this was unlikely to be significant as few were excluded for these reasons. Our findings from children who were HIV negative with mostly mild disease may not be generalizable to settings where presentations with advanced disease and HIV are common. Furthermore, this hypothesis-driven transcriptomic approach misses unknown mediators that may be important in systemic inflammation in TBM. We did not have the scope to study MMP concentrations in CSF and investigate whether gene expression in whole blood reflects disease on a protein level in the CNS. Future validation studies with longitudinal sampling in different cohorts should incorporate CSF cytokine and protein confirmation of transcriptomic signals, especially MMP-8. Study drug treatment allocations may influence results, especially where the sample size is small. Study strengths are the in-depth characterization of CNS lesions and clinical features including extraneural disease. Integration with transcriptomic analyses allowed for the unique opportunity to evaluate how specific inflammatory mediators influence disease phenotype. This exploratory study is an important initial step to better understanding the mechanisms of cerebral ischemia in TBM and directing research on host-directed therapy to improve outcomes from this devastating disease.

## Supplementary Material

jiaf399_Supplementary_Data

## Supplementary Data

Supplementary materials are available at *The Journal of Infectious Diseases* online (http://jid.oxfordjournals.org/). Supplementary materials consist of data provided by the author that are published to benefit the reader. The posted materials are not copyedited. The contents of all supplementary data are the sole responsibility of the authors. Questions or messages regarding errors should be addressed to the author.
