# Supplementary material for: Brain Imaging and Whole Blood Targeted Transcriptomic Analyses to Characterize Cerebral Infarctions in Children With Tuberculous Meningitis
Source: J Infect Dis. 2025 Aug 2;232(4):e676–81. doi: 10.1093/infdis/jiaf399 (PMC12526924; doi:10.1093/infdis/jiaf399)
Supplement: jiaf399_Supplementary_Data [file jiaf399_supplementary_data.docx]

**Supplementary material**

**Table S1** Definition of refined British medical research council grades

**Table S2** Age and disease severity of children who consented but could not have brain MRI imaging

**Table S3** Age groups of children with definite, probable, possible TBM and those who had Whole blood RNA sequencing

**Table S4** Clinical risk factors for TBM-associated cerebral infarcts

**Figure S1** Expression of selected inflammatory mediators in whole blood of TBM infected children with hydrocephalus

**Figure S2** Expression of selected inflammatory mediators in whole blood of TBM infected children with tuberculomas

**Figure S3** Expression of selected inflammatory mediators in whole blood of TBM infected children with extra-neural TB

**Figure S1 Alt text**

Box plots and data depicting gene expression categorized by absence or presence of cerebral infarctions in children with TBM. Each coloured panel is labelled according to the selected gene (10 genes in total).

**Figure S2 Alt text**

Box plots and data depicting gene expression categorized by absence or presence of hydrocephalus in children with TBM. Each coloured panel is labelled according to the gene (10 selected genes in total).

**Figure S3 Alt text**

Box plots and data depicting gene expression categorized by absence or presence of CNS tuberculomas in children with TBM. Each coloured panel is labelled according to the selected gene (10 selected genes in total).

**Table S1**

**Refined British medical research council grades**

| **Grade** | **Definition** |
| --- | --- |
| I | GCS = 15 and no focal neurology |
| IIa | GCS 15 with focal neurology |
| IIb | GCS 11-14 with or without focal neurology |
| III | GCS < 11 with or without focal neurology |

*Van Toorn R et al. Int J Tuberc Lung Dis. 16(5): 628-632*

| **Participant No.**  **Table S2** | **Age (years)** | **MRC Grade** | **Reason for exclusion** |
| --- | --- | --- | --- |
| 1 | 1.7 | 1 | COVID-19 restrictions & limited imaging facilities |
| 2 | 1.8 | 2 | COVID-19 restrictions & limited imaging facilities |
| 3 | 9.5 | 2 | COVID-19 restrictions & limited imaging facilities |
| 4 | 0.3 | 1 | Excessive movement |
| 5 | 0.4 | 1 | Outside time window of MRI protocol |
| 6 | 0.3 | 1 | Excessive movement |
| 7 | 10.2 | 1 | Alternative diagnosis |
| 8 | 0.2 | 2 | Excessive movement |
| 9 | 1.5 | 1 | Outside time window of MRI protocol |
| 10 | 0.3 | 1 | Outside time window of MRI protocol |
| 11 | 0.5 | 1 | Excessive movement |
| 12 | 12.6 | 3 | Death before imaging |
| 13 | 0.7 | 2 | Excessive movement |
| 14 | 4.1 | 1 | Excessive movement |
| 15 | 3.2 | 1 | Excessive movement |
| 16 | 0.2 | 2 | Excessive movement |
| 17 | 1.1 | 3 | Too medically unstable/MRI impracticable |
| 18 | 9.7 | 2 | Death before imaging |
| 19 | 0.7 | 2 | Excessive movement |
| 20 | 4.1 | 1 | Excessive movement |
| 21 | 3.2 | 1 | Excessive movement |
| 22 | 0.2 | 2 | Excessive movement |
| 23 | 0.5 | 1 | Excessive movement |
| 24 | 7.8 | 2 | Too medically unstable/MRI impracticable |
| 25  MRC = medical research council, MRI = magnetic reasonance imaging, COVID-19 = coronavirus disease 2019  j  **Table 1. RNA sequencing and TBM diagnosis by Age groups**   \|  \| \| \| **Age Groups** \| \| \| \|  \| \| --- \| --- \| --- \| --- \| --- \| --- \| --- \| --- \| \| **Variable** \| **N** \| **Overall**  N = 30^1^ \| **0-<2**  N = 7^1^ \| **2-<5**  N = 4^1^ \| **5-<11**  N = 4^1^ \| **>=11**  N = 15^1^ \| **p-value**^2^ \| \| **RNA sequencing** \| 30 \| 19 (63%) \| 6 (86%) \| 3 (75%) \| 3 (75%) \| 7 (47%) \| 0.350 \| \| **TBM diagnosis** \| 30 \|  \|  \|  \|  \|  \| 0.248 \| \| *Definite* \|  \| 12 (40%) \| 2 (29%) \| 2 (50%) \| 1 (25%) \| 7 (47%) \|  \| \| *Probable* \|  \| 7 (23%) \| 2 (29%) \| 2 (50%) \| 2 (50%) \| 1 (6.7%) \|  \| \| *Possible* \|  \| 11 (37%) \| 3 (43%) \| 0 (0%) \| 1 (25%) \| 7 (47%) \|  \| \| ^1^Frequency (%) \| \| \| \| \| \| \| \| \| ^2^Fisher's exact test \| \| \| \| \| \| \| \| | 0.5 | 2 | Too medically unstable/MRI impracticable |

|  | | | **Age Groups** | | | |  |
| --- | --- | --- | --- | --- | --- | --- | --- |
|  | **N** | **Overall**  N = 30^1^ | **0 - <2**  N = 7^1^ | **2 - < 5**  N = 4^1^ | **5 - < 11**  N = 4^1^ | **> = 11**  N = 15^1^ | **p-value**^2^ |
| **RNA sequencing** | 30 | 19 (63%) | 6 (86%) | 3 (75%) | 3 (75%) | 7 (47%) | 0.350 |
| **TBM diagnosis** | 30 |  |  |  |  |  | 0.248 |
| *Definite* |  | 12 (40%) | 2 (29%) | 2 (50%) | 1 (25%) | 7 (47%) |  |
| *Probable* |  | 7 (23%) | 2 (29%) | 2 (50%) | 2 (50%) | 1 (6.7%) |  |
| *Possible* |  | 11 (37%) | 3 (43%) | 0 (0%) | 1 (25%) | 7 (47%) |  |
| ^1^Frequency (%) | | | | | | | |
| ^2^Fisher's exact test | | | | | | | |

**Table S3**

| **Table S4** | | | **Infarct** | |  |
| --- | --- | --- | --- | --- | --- |
|  | **N** | **Overall**  N = 30^1^ | **No**  N = 11^1^ | **Yes**  N = 19^1^ | **p-value**^2^ |
| **Age (years)** | 30 | 11.0 (2.4, 13.2) | 12.4 (2.7, 13.4) | 9.5 (1.5, 12.3) | 0.216 |
| **Duration of fever** | 30 | 10 (7, 19) | 8 (3, 20) | 10 (7, 19) | 0.682 |
| **Convulsions** | 30 |  |  |  | 0.129 |
| No |  | 25 (83.3%) | 11 (100.0%) | 14 (73.7%) |  |
| Yes |  | 5 (16.7%) | 0 (0.0%) | 5 (26.3%) |  |
| **Glascow coma scale** | 30 |  |  |  | >0.999 |
| 8 |  | 1 (3.3%) | 0 (0.0%) | 1 (5.3%) |  |
| 14 |  | 1 (3.3%) | 0 (0.0%) | 1 (5.3%) |  |
| 15 |  | 28 (93.3%) | 11 (100.0%) | 17 (89.5%) |  |
| **Focal neurology** | 30 |  |  |  | 0.372 |
| No |  | 24 (80.0%) | 10 (90.9%) | 14 (73.7%) |  |
| Yes |  | 6 (20.0%) | 1 (9.1%) | 5 (26.3%) |  |
| **TBM grade*** | 30 |  |  |  | 0.520 |
| mild disease |  | 28 (93.3%) | 11 (100.0%) | 17 (89.5%) |  |
| moderate-severe disease |  | 2 (6.7%) | 0 (0.0%) | 2 (10.5%) |  |
| **CSF parameters** |  |  |  |  |  |
| protein | 30 | 1.2 (0.9, 1.9) | 1.1 (0.4, 1.3) | 1.7 (1.0, 2.1) | **0.045** |
| glucose | 30 | 2.0 (1.6, 2.7) | 2.4 (2.0, 2.7) | 1.8 (1.4, 2.4) | **0.023** |
| neutrophil (cells/ul) | 27 | 0.0 (0.0, 27.0) | 2.5 (0.0, 10.0) | 0.0 (0.0, 27.0) | 0.914 |
| *(Missing)* |  | 3 | 1 | 2 |  |
| lymphocyte (cells/ul) | 30 | 96.2 (46.0, 247.9) | 208.8 (30.0, 807.5) | 92.4 (46.0, 155.8) | 0.478 |
| **Blood parameters** |  |  |  |  |  |
| Neutrophil  (10^3^ cells/ul) | 30 | 6.0 (4.4, 8.6) | 5.1 (4.7, 8.6) | 6.3 (4.3, 11.0) | 0.703 |
| Lymphocytes  (10^3^ cells/ul) | 30 | 2.3 (1.9, 4.4) | 2.7 (2.1, 5.9) | 2.2 (1.8, 4.4) | 0.287 |
| Neut:Lymph ratio | 30 | 2.2 (1.4, 4.5) | 2.1 (1.2, 2.2) | 3.2 (1.4, 4.9) | 0.232 |
| Platelet  (10^3^ cells/ul) | 30 | 402 (369, 523) | 392 (263, 456) | 444 (379, 531) | 0.263 |
| **Extra-neural TB** | 30 |  |  |  | 0.672 |
| No |  | 22 (73.3%) | 9 (81.8%) | 13 (68.4%) |  |
| Yes |  | 8 (26.7%) | 2 (18.2%) | 6 (31.6%) |  |
| **Hydrocephalus** | 30 |  |  |  | 0.063 |
| No |  | 12 (40.0%) | 7 (63.6%) | 5 (26.3%) |  |
| Yes |  | 18 (60.0%) | 4 (36.4%) | 14 (73.7%) |  |
| **Tuberculomas** | 30 |  |  |  | >0.999 |
| No |  | 20 (66.7%) | 7 (63.6%) | 13 (68.4%) |  |
| Yes |  | 10 (33.3%) | 4 (36.4%) | 6 (31.6%) |  |
| **Basal meningeal enhancement** | 30 |  |  |  | 0.129 |
| No |  | 25 (83.3%) | 11 (100.0%) | 14 (73.7%) |  |
| Yes |  | 5 (16.7%) | 0 (0.0%) | 5 (26.3%) |  |
| ^1^Median (Quantile 1, Quantile 3) or Frequency (%) | | | | | |
| ^2^Wilcoxon rank sum exact test; Wilcoxon rank sum test; Fisher's exact test | | | | | |
| *TBM grade mild = Grade I and Grade IIa, moderate – severe Grade IIb and Grade III | | | | | |


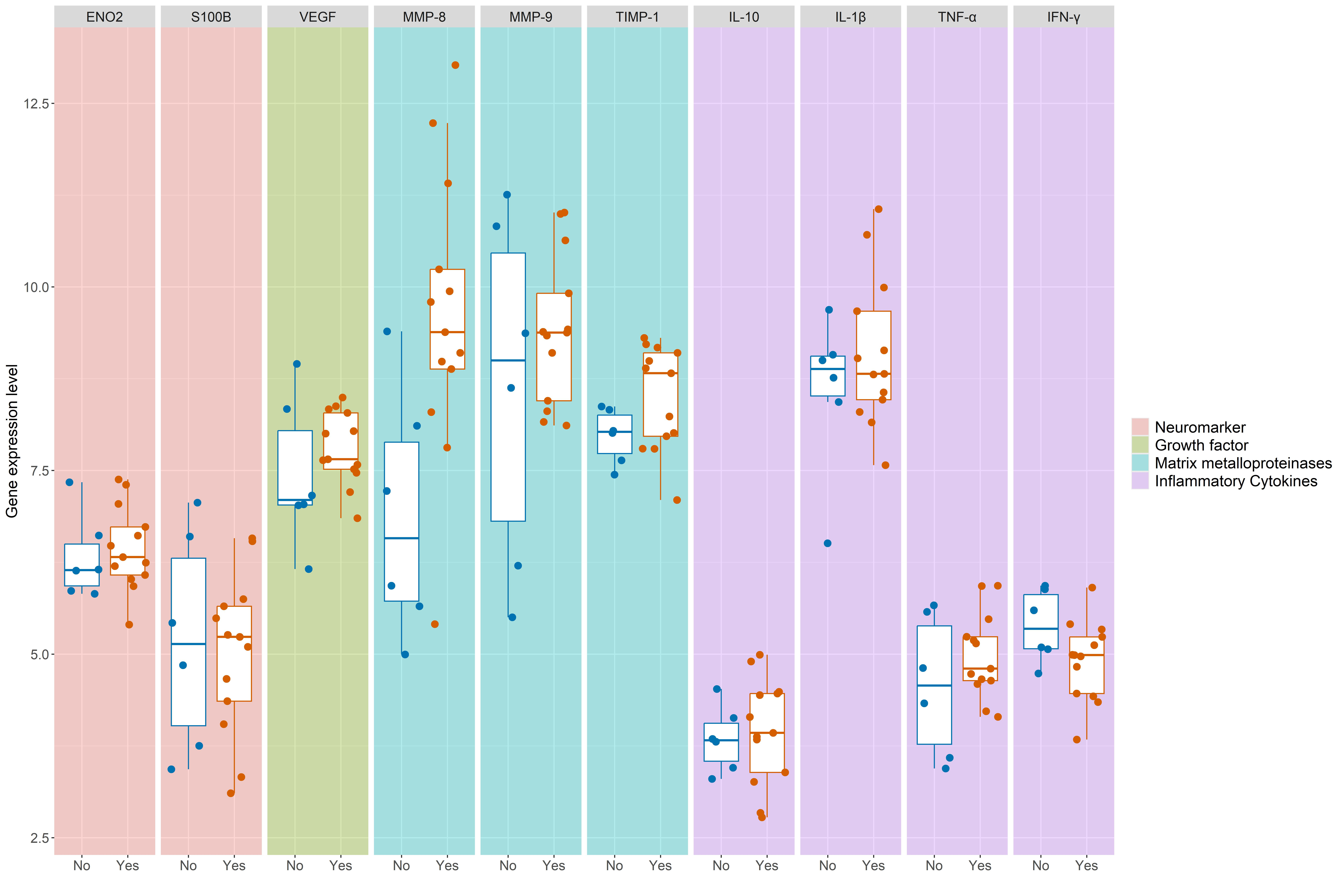


**Figure S1. Expression of selected inflammatory mediators in whole blood of TBM infected children with hydrocephalus**

N=19 children with definite/probable/possible TBM had baseline MRI and whole blood RNA sequencing for neuromarkers in the pink panel (ENO2, S100B), inflammatory cytokines in the purple panel (IL-1b, IFN-g, TNF-a, IL-10), growth factors (VEGF) in the green panel and Matrix metalloproteinases in the blue panel (MMP-8, MMP-9, TIMP-1). N = 13 children with hydrocephalus compared to N = 6 those without. Dots represent individual patient data, horizontal lines represent median, Q1 and Q3.


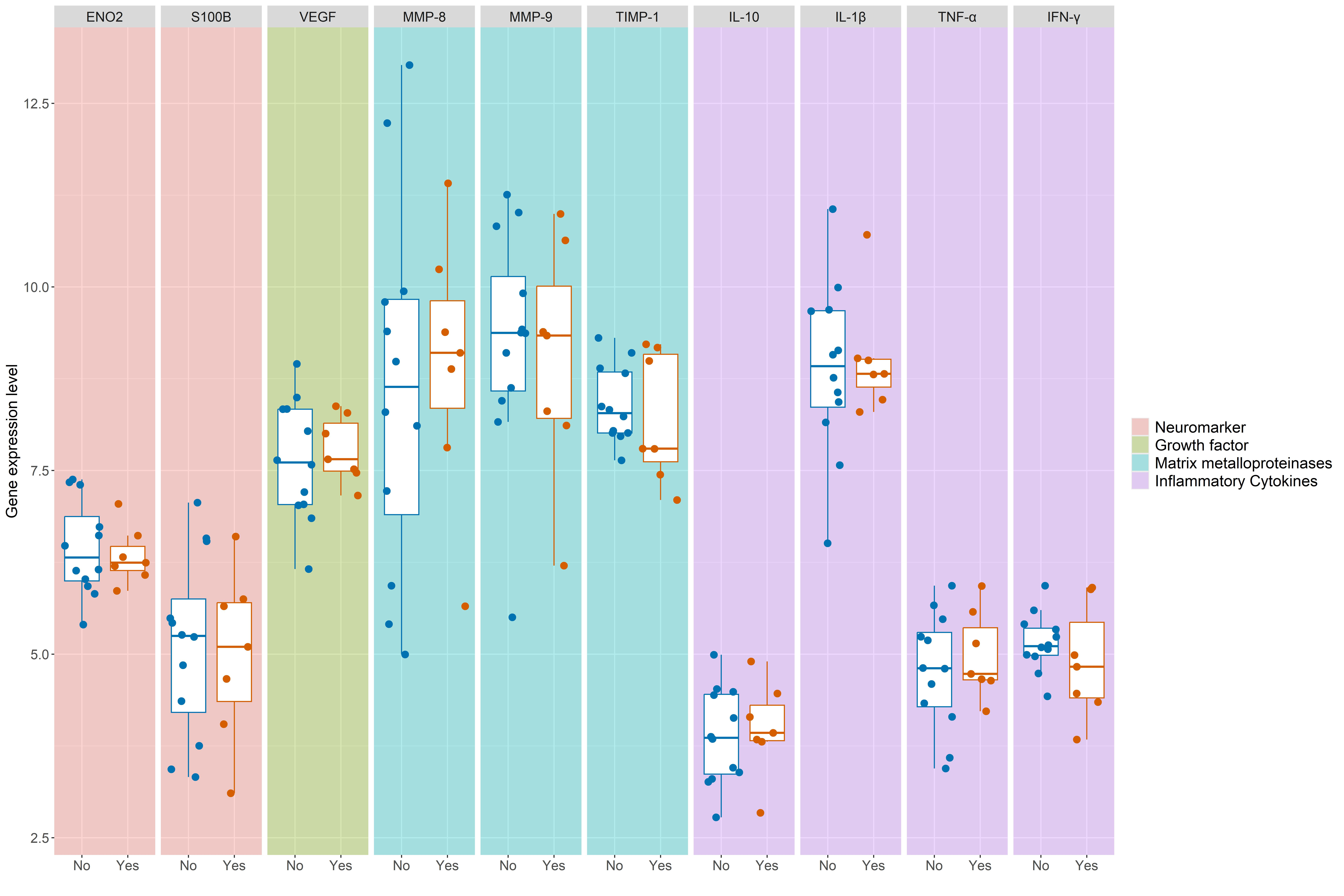


**Figure S2. Expression of selected inflammatory mediators in whole blood of TBM infected children with tuberculomas**

N=19 children with definite/probable/possible TBM had baseline MRI and whole blood RNA sequencing for neuromarkers in the pink panel (ENO2, S100B), inflammatory cytokines in the purple panel (IL-1b, IFN-g, TNF-a, IL-10), growth factors (VEGF) in the green panel and Matrix metalloproteinases in the blue panel (MMP-8, MMP-9, TIMP-1). N = 7 children with CNS tuberculomas comapred to N = 12 children without. Dots represent individual patient data, horizontal lines represent median, Q1 and Q3.


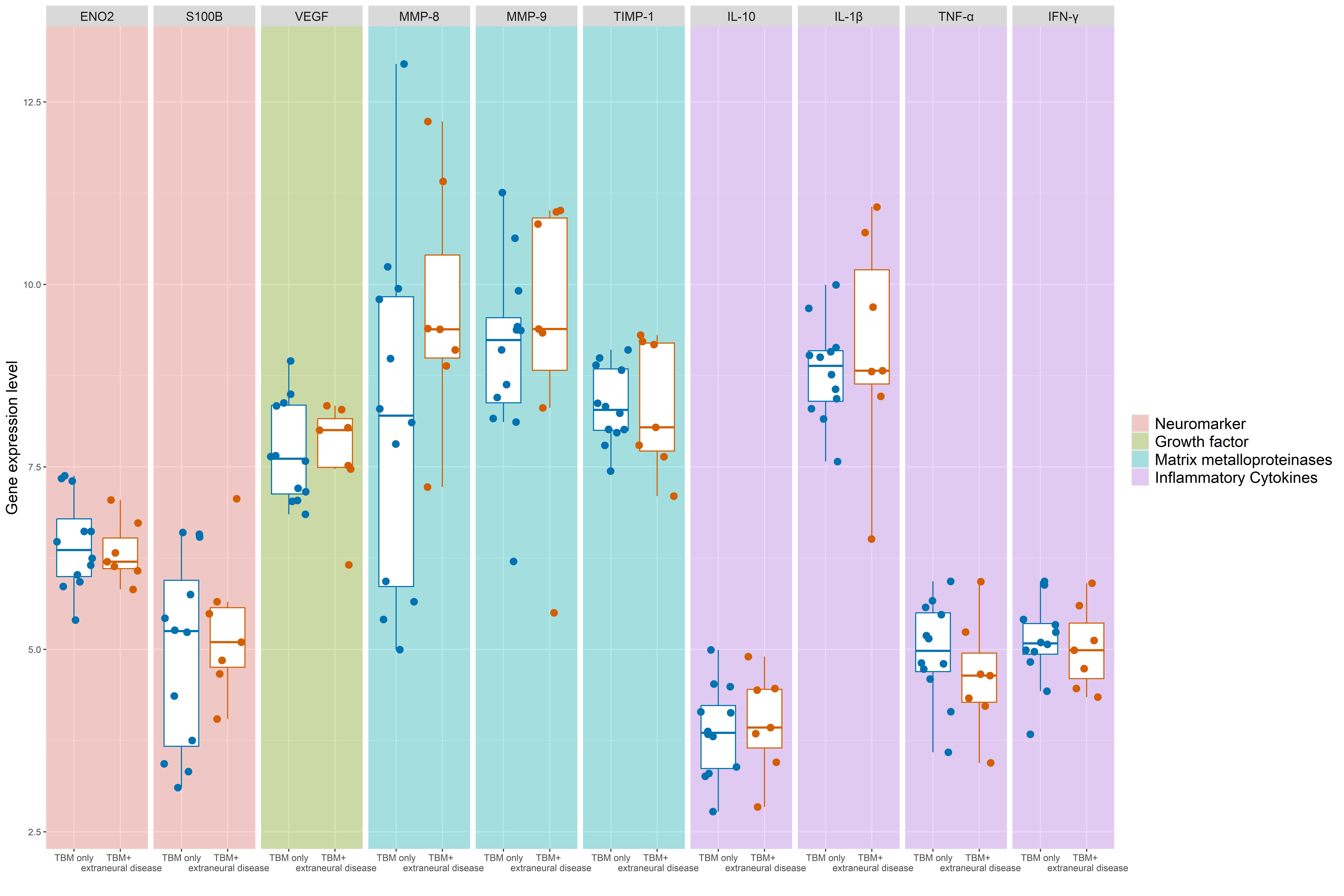


**Figure S3. Expression of selected inflammatory mediators in whole blood of TBM infected children with extra-neural TB**

N=19 children with definite/probable/possible TBM had baseline MRI and whole blood RNA sequencing for neuromarkers in the pink panel (ENO2, S100B), inflammatory cytokines in the purple panel (IL-1b, IFN-g, TNF-a, IL-10), growth factors (VEGF) in the green panel and Matrix metalloproteinases in the blue panel (MMP-8, MMP-9, TIMP-1). N = 7 children with concomitant extra-neural TB compared to N = 12 children with TBM only. Dots represent individual patient data, horizontal lines represent median, Q1 and Q3.
